# Plant growth–promoting microbes and microalgae-based biostimulants: sustainable strategy for agriculture and abiotic stress resilience

**DOI:** 10.1098/rstb.2024.0251

**Published:** 2025-05-29

**Authors:** Cristina Brito-Lopez, Nicky van der Wielen, Maria Barbosa, Rumyana Karlova

**Affiliations:** ^1^ Laboratory of Plant Physiology, Plant Sciences Group, Wageningen Universiteit en Research, Wageningen, Gelderland 6708 PB, The Netherlands; ^2^ Bioprocess Engineering, Wageningen Universiteit en Research, Wageningen, Gelderland 6708 PB, The Netherlands

**Keywords:** biostimulants, stress resilience, drought, PGPM, microalgae

## Abstract

Climate change events significantly impact the food production chain by damaging crops in their most fragile phenological states. Furthermore, increasing human population and excess food waste present agricultural systems with the challenge of closing the yield gap and securing food demands in the future as well as protect the soil health and biodiversity. Biostimulants are a novel alternative in agriculture that can effectively use inputs, enhance crop resilience to abiotic stresses and improve food quality. Additionally, biostimulants offer a promising and eco-friendly solution for reducing the use of chemical fertilizers, as they have the potential to increase crop nutrient use efficiency and yield. Because of their effects on plant growth, a wide range of products can be marketed as biostimulants. Presented in this review is an overview of recent literature on the use of plant growth-promoting microbes and microalgae-derived extracts obtained from either waste streams or recycled substrates. Starting from their source material, extraction technologies and application modalities, a view of their factors shaping the composition and activity of biostimulants is provided to elucidate a mechanistic model of action which leads to increased stress resilience in crops. This work further sets out to understand if the biostimulants can be used to transform waste into a valuable product that can accelerate the transition to sustainable agriculture.

This article is part of the theme issue ‘Crops under stress: can we mitigate the impacts of climate change on agriculture and launch the ‘Resilience Revolution’?’.

## 1. Introduction

The impact of global warming has become increasingly evident through rising temperatures, prolonged droughts and increased soil salinity worldwide [1,2]. These abiotic factors can have a severe impact on crop yields and agricultural outputs [2,3]. As the global population continues to grow, agricultural productivity must increase proportionally to meet rising demands and close the future yield gap [1,4]. Furthermore, it is predicted that environmental stresses such as heat, drought and salinity, caused by global climate change, will lead to a 50% reduction of arable areas by 2050 [5]. Addressing these challenges requires a shift in agro-ecosystem management.

Traditional use of chemical fertilizers contributes to environmental pollution and accelerates soil salinization, highlighting the urgent need for eco-friendly and sustainable innovative solutions [6]. Implementing new approaches allowing crops to be grown in saline conditions could have a crucial impact on agriculture in general, allowing for the valorization of non-agricultural land and brackish water. One promising alternative solution involves the use of biostimulants, which can use the interaction between plants and soil ecosystems.

Biostimulants are defined as organic substances or microorganisms that could increase quality parameters by improving nutrient and water use efficiency of crops as well as their resilience to abiotic stresses [7]. They are categorized by their origin or active ingredients into three types: microbial, non-microbial and waste-derived [8]. While microbial biostimulants comprise plant growth-promoting microbes (PGPM) that are naturally found in the rhizosphere, the non-microbial and waste-derived types contain organic substances. They are extracted from many sources including underused biomass from food and agricultural industries which in turn contribute to sustainable agriculture [9]. The recruitment of PGPM enhances the interactions between the soil microbiome and crops, boosting plant resilience and supporting plant growth [10,11]. Plant biostimulants are applied through soil drenching or foliar spraying at low concentrations, which differentiates their mode of action from synthetic inorganic fertilizers [12].

In this review, we elucidate the composition, production and mode of action of microbial and microalgae crude extracts biostimulants as a new sustainable approach. Additionally, we discuss the opportunities and challenges for level-up sustainable production and their implementation as complementary practice in plant nutrition management to improve plant performance.

## 2. Composition of biostimulants is key to their function

### (a) Factors shaping the composition and activity of biostimulants

Biostimulants are divided into three categories: microbial, non-microbial and waste-derived [8]. Both PGPM, microalgae and cyanobacteria are capable of adapting to harsh environments by producing a wide array of diverse metabolites. They are a rich source of diverse bioactive molecules, which have been explored for various purposes such as food, feed and fuel over several decades [13]. These metabolites are known for activating several mechanisms in plants, such as nutrient uptake, secondary metabolite pathways and abiotic stress mitigation [14,15]. Among the compounds found in biostimulants, phytohormones, hormone-like substances, proteins, peptides and amino acids, fatty acids, poly- and oligosaccharides and phenolic compounds have demonstrated potential plant biostimulatory effects [16]. However, the presence and concentrations of these active compounds will depend strongly on the composition (species used) and formulation (methods used) for the production of biostimulants.

### (b) Composition of plant growth-promoting microbes and algae biostimulants

Microbes are natural inhabitants of the soil and have evolved alongside plants for millions of years [17,18]. Their biodiversity and functionality have declined owing to the prevalence of monocultural agriculture and chemical pollution [19–22]. Currently, there is growing attention to incorporate PGPM as biostimulants and integrating them as a widespread agricultural practice, despite many challenges that still need to be addressed [23].

Microbes are the major component in microbial biostimulants, which can be composed of one specific organism or a consortium of microbes, albeit inter- or intra-kingdom [24,25]. The most popular choices for selection of microbial biostimulants are arbuscular mycorrhizal fungi (AMF) and plant growth-promoting rhizobacteria (PGPR), which actively colonize the rhizosphere. AMF belong to the phylum Glomeromycota which can colonize roots of more than 80% of land plants [26]. Commercial inoculants often contain AMF belonging to the genera Rhizophagus and Funneliformis owing to their widespread occurrence in soils and broad host compatibility [27]. Besides AMF, Trichoderma and Gliocladium are fungal taxa commonly used in biostimulants [28].

PGPR represents a highly diverse group comprising numerous genera from the phyla Proteobacteria, Firmicutes, Actinobacteria and Bacteroidetes. These bacteria are involved in a wide range of functions that promote plant health [29]. Among these phyla, genera such as a *Aeromonas, Arthrobacter, Azospirillum, Azotobacter, Bacillus, Enterobacter, Flavobacterium, Burkholderia, Klebsiella, Pseudomonas, Rhizobium, Serratia* and *Streptomyces* belong to the most studied PGPR and are the most prominent bacteria present in microbial biostimulants [27,30].

The right content and composition of a PGPM-based biostimulant is not universal but rather determined by the purpose of its use and its suitability for the site of application [7]. Microbial biostimulants can restore soil health, promote plant growth in a wide range of crops or target a particular challenge during crop cultivation [31–34]. Their composition can be tailored to achieve various objectives such as improving nutrient uptake, increasing stress resilience, providing biocontrol and enhancing crop quality [30,35]. The environmental conditions in which the biostimulant is applied further shape its final bioactive composition, as factors such as soil type, history of cultivation, crop species and native microbial communities all influence the choice of PGPMs [36]. Additional compounds in the formulation, designed to maintain microbial viability and encourage their establishment, can further enhance the plant growth-promoting effects [27].

Studies have shown that inter-kingdom and microbe blends are more effective than using single-strain inoculants [24,37]. Nevertheless, despite that a consortium of microbes has been shown to be more effective, blending microbes for biostimulants is neither straightforward nor guaranteed to have an additive effect [25,38]. To improve the effectiveness of the consortia, compatibility testing is essential. Native microbes were shown to establish and perform better than non-native ones, probably owing to their adaptation to the local environment [27,39]. A deeper understanding of the underlying mechanisms of microbial communication will enhance predictability, simplifying the development of biostimulants with desired properties.

Each field, crop and goal may need a unique blend, positioning microbial biostimulants as a highly specialized product. The variations in their composition require different sets of microbes, as well as distinct culturing methods and formulations [36,40]. As a result, microbial biostimulants can become too specialized, reducing their attractiveness for large-scale use and commercialization. However, the function of the biostimulant remains the primary criterion in selecting which PGPM will be used in the formulation. To maximize their potential, it is crucial to develop versatile and broadly applicable formulations that meet diverse agricultural needs [25].

Unlike microbial biostimulants, which colonize the rhizosphere, microalgae are commonly used in the form of crude extracts that use the bioactive components produced during their growth phase. Although the biostimulant activity of these extracts remains uncertain, their application to plants enhances quality and fertility of the soil and improves the availability, uptake and use of nutrients [4,41]. By fostering a beneficial soil microbiome and promoting enzymatic activity, they also indirectly contribute to soil improvement, thereby maintaining soil quality and making essential nutrients available for optimal plant growth [42,43]. The synergistic effects of different compounds within the crude extracts may also explain the plant growth stimulation [16].

Additionally, algae are an attractive source for biostimulant use in agriculture owing to their metabolic profile and ecological benefits [44]. Algae biomass, comprising both seaweed and microalgae, represents a promising and sustainable source as feedstock for diverse products from biofuels to biostimulants [45,46]. Seaweed has been used in agriculture for hundreds of years and has been comprehensively reviewed as a plant biostimulant over the last decades [47]. Both seaweed and microalgae are sources of valuable components that boost crop production and improve stress resilience [4,16]. However, given the extensive coverage of seaweed in existing literature, this review focuses exclusively on microalgae as a novel source of both microbial (cyanobacteria) and non-microbial (microalgae crude extracts) biostimulants in sustainable agriculture.

While the production of microalgae is still developing, only a few genera are industrially produced and exploited for the biostimulant industry. *Arthrospira, Chlorella, Dunaliella* and *Scenedesmus* are the most commonly used genera [16], highly exploited for their rapid growth rates [48]. This limitation in the number of genera may be attributed to the strict regulations for the exploration and production of these strains for both human consumption and agricultural applications.

The biorefinery of microalgae presents a significant challenge to produce biostimulants. The processes of harvesting, cell disruption and compound extraction require extensive optimization. Various approaches, including mechanical, chemical and enzymatic methods, must be carefully evaluated [16]. The selection of the most appropriate technique should be based on the specific compound(s) of interest, as some methods may not be suitable for extracting certain compounds while preserving their bioactivity. For instance, some mechanical techniques can increase the temperature of the crude extract, affecting the bioactivity of sensitive compounds that might be involved in the biostimulatory effect. Lately, novel nanotechnological non-destructive approaches are being explored for extracting sensitive compounds from microalgae [49]. However, identifying the active components in microalgae that are beneficial for agriculture is essential. This knowledge could allow us to repurpose the non-active components for other applications and thereby increase the sustainability of the process.

### (c) Sustainable production of biostimulants

To reach an industrial level of biostimulant production, both microbes and microalgae could be cultured through targeted culturing methods taking place in bioreactors. Their cultivation in bioreactors significantly enhances land use efficiency, as it eliminates the need for arable land in their production [50]. A sustainable approach would involve using carbon sources from food and agro-industrial organic waste and by-products using continuous and renewable waste products available in sufficient quantities at minimal cost [21,50–53]. This approach aligns perfectly with the principles of a circular economy, where waste is minimized, resources are continuously reused and recycled and environmental pollution is reduced.

Regarding microbes, stable manure, livestock slurry, derived compost, industrial cooling systems or scrubber water from air washers in livestock and compost facilities are common waste products that could serve as carbon sources or carriers for microbial inoculants [21,54–57]. High-nutrient wastewater also provides an ideal environment for the growth of both nitrifying bacteria and microalgae [58,59]. Using the ability of these microorganisms to recover nutrients and produce biostimulants presents a promising opportunity to implement a circular approach in the system. Although further research is needed for large-scale implementation, using these abundant waste products offers a sustainable source for biostimulant production.

Developing microbial biostimulants starts with the isolation and screening for potential candidates, followed by field evaluation of their efficacy and ending with large-scale production and commercialization (36). However, viability during large-scale culturing, stability in formulations and retaining its plant growth-promoting effect within the native soil microbial community largely decrease the list of potential microbes as biostimulants [60–62]. In addition, since products containing live organisms are often heterogeneous and root colonization efficiency is dependent on numerous factors, there are many inconsistencies when tested in the field [28,36,63]. Therefore, it is important to develop effective formulations with the right carrier and application methods to ensure the reliability of microbial biostimulants.

Current European Union regulations for microbial biostimulant composition are restrictive, only allowing four genera: *Azotobacter* spp., Mycorrhizal fungi, *Rhizobium* spp. and *Azospirillum* spp*.* [*64* 64]. These tight regulations could be revised in the future to include more potential candidates as biostimulants, allowing further advancement of this field [27]. Biostimulants are great candidates to boost crop productivity, improve plant and soil health and at the same time support sustainability efforts. Therefore, legislation should be more accommodating to encourage such advancements.

While microalgae are relatively easy to cultivate, reducing freshwater input is still a crucial factor for improving sustainability. The ability of microalgae to thrive in wastewater environments is especially appealing owing to their capability to convert chemical contaminants into biomass [65]. Moreover, these microorganisms have been used for nutrient recovery and purification of waste streams as a well-established strategy that mitigates the environmental impact of waste since the 1970s [50,66].

**Figure 1.**
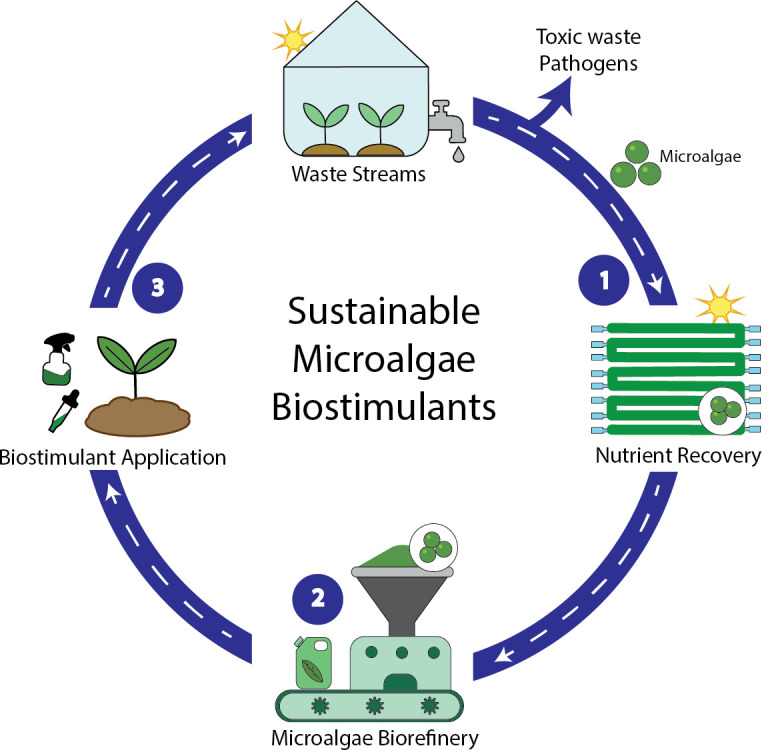
Sustainable biostimulant production is driven by three main stages: (1) nutrient recovery from waste streams; (2) optimization of biostimulant production using a biorefinery approach; and (3) optimization of dosage and type of application of biostimulants in crops.

Combining the remarkable ability of microalgae to recover nutrients with their potential to convert these nutrients into high-value compounds results in significant environmental and economic benefits. Thus, achieving a more sustainable production for microalgae biostimulants involves three key stages (figure 1): (i) nutrient recovery, (ii) conversion of the raw sludge into biostimulants, and (iii) application of the bioproduct in crop production.

Many strains of microalgae have exhibited efficient nutrient removal from wastewater exceeding 70% and surpassing 90% for nitrates, phosphates, organic carbon, ammonia and others, acting as natural filters and bringing great advantages compared to traditional wastewater treatments [67–69]. Therefore, wastewater from various industries, including piggery, domestic, brewery, paper-pulp and greenhouse operations, has been used as a nutrient source for microalgae production to meet discharge level requirements [16,67]. Besides mitigation of carbon emissions, the significant benefit from using microalgae as natural filters is the production of a versatile resource that offers multiple applications including the production of biostimulants [70].

Although microalgae are highly efficient at removing nutrients from wastewater, several limitations can impact biomass composition and therefore biostimulant production. Growing microalgae in wastewater can lead to nutrient imbalances, as industries do not consistently discharge the same volume of water at the same nutrient concentration, affecting the influx for microalgae growth. For instance, high-strength wastewater such as in the piggery sector requires an initial dilution of the waste stream with fresh water and subsequent recirculation of the purified water to achieve more suitable growth conditions [71]. Nutrient instability can influence biomass composition, as the production of valuable components in microalgae depends on the growth conditions. For example, the accumulation of proteins, lipids and carbohydrates in many microalgae strains is triggered by nitrogen (N) starvation, changes in light intensity or salinity stress conditions [72,73].

Furthermore, the efficient absorption of heavy metals by microalgae [74] presents an extra challenge for using the resulting biomass as feedstock in biostimulant production. According to the European Parliament and Council Regulation (European Union) 2019/1009, biostimulants must not contain toxic elements. Although there is no evidence of the effects of using biomass containing heavy metals in crop production, small amounts of these toxic elements could pose a risk to human health [75]. The subsequent use of this ‘low-quality biomass’ can also be repurposed in many other industries, including biofuel production or electricity generation. Nevertheless, microalgae are excellent candidates for nutrient recovery, providing a solution that is both environmentally friendly and highly sustainable.

Once the nutrients have been converted into high-value biomass, transforming the raw sludge into biostimulants through cost-effective and low-pollution processes becomes the primary focus of emerging biostimulant companies. However, since the activity of biostimulants can vary at each step of the process, the current production aims to obtain a ‘smoothie-like’ crude extract containing most components resulting from the eco-friendly disruption process. This crude extract contains mainly proteins (30–70%), carbohydrates and lipids (30–40%), with amounts varying depending on the strain and intensity of the processes. Less abundant bioactive compounds are also part of the composition but might be lost owing to degradation or damage during processing [76]. Identifying a process that is compatible with sensitive molecules involved in plant responses is crucial, as one of the main challenges in biostimulant production is achieving a stable formulation.

The diversity and complex composition of microalgae present an additional challenge. With a wide range of species, each having unique characteristics, not all microalgae can be processed using the same method. For instance, strains like *Chlorella* sp. or *Tetradesmus* sp. which have shown effective nutrient recovery and biostimulatory effect in several plant species [16,77], feature thick and robust cell walls hindering harvest optimization. Both traditional and modern techniques for releasing components from microalgae cells have been employed to retain the majority of the components in an active state [49,78]. Mechanical, chemical and enzymatic disruption are the most popular techniques used at pilot-scale and industrial level to achieve maximal release of bioactive components [49]. Acid hydrolysis has been widely used for the production of biostimulants at an industrial scale owing to its effectiveness. However, mechanical disruption methods, such as high-pressure homogenizers, produce comparable results of the biostimulatory activity and are therefore prioritized over chemical and enzymatic methods owing to sustainability concerns [79].

In recent years, the biostimulatory activity of microalgae and PGPMs on a diverse range of crops has been extensively documented [16,35,41]. However, their biostimulant effects are not consistent and reproducible. Several factors can influence the effectiveness of biostimulants, including microalgae/PGPMs strain, cultivation conditions, processing methods, dosage and field application [63]. For PGPM, once the mechanisms underlying the microbial biostimulant effect are understood, developing cell-free formulations or incorporating specific bioactive compounds derived from PGPM into biostimulants could simplify the application process [63]. This approach would also help to address challenges related to the stability and consistency of microbial biostimulants [62,80].

Increasing efforts are being made to not only regulate but also standardize parameters throughout the entire production process of biostimulants. More stakeholders, including policymakers and scientists, recognize the need to implement new guiding principles to substantiate plant biostimulant claims [81]. Therefore, to enhance marketability of biostimulants and bridge the gap between science and crop production, it is essential to demonstrate their effectiveness. Identifying the active components in microalgae that are beneficial for agriculture is crucial. This knowledge could allow us to repurpose the non-active components for other applications in order to optimize the product and to aim at zero waste products. For instance, if polysaccharides (PSs) are found to trigger plant responses, other valuable components like pigments can be extracted beforehand and be used in different sectors. Furthermore, rigorous quality control measures in composition and production of biostimulants are essential to ensure the consistency and efficacy of the final biostimulant products. These measures are also crucial for establishing biostimulants as a common practice among farmers, ultimately enhancing crop production.

## 3. The plant growth-promoting effect of biostimulants

PGPM have shown to improve phenotypic traits, leading to increased biomass and improved crop yield [43,82,83]. Studies have shown a correlation between the presence of PGPM and enhanced plant growth, increased leaf N content, higher yield, improved protein content and extended shelf life across different crops [32,33,84]. Additionally, under stress conditions, plants inoculated with PGPM have shown higher tolerance compared to the uninoculated control [30,36]. However, the mechanisms by which PGPM influence plant growth is physiologically diverse and remain largely undefined.

**Figure 2.**
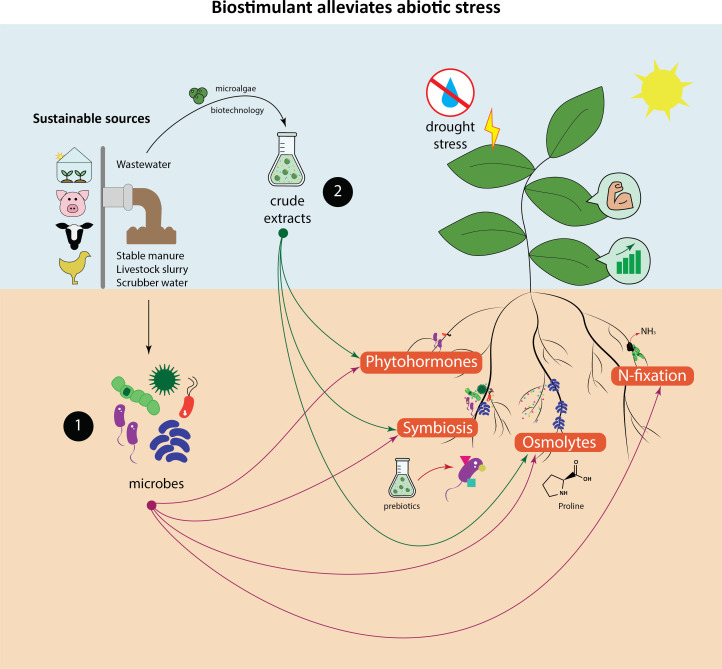
Recycled waste is a sustainable source for the production of (1) microbial and (2) non-microbial biostimulants. They alleviate abiotic stresses like drought through production of phytohormones, enhancing symbiosis, osmolytes accumulation and N fixation.

The biostimulatory effect of microbes, including microalgae and cyanobacteria, functions both directly and indirectly. The direct effect of them arises from their presence and corresponding metabolic activity in the soil, while the indirect pathway involves enhancing plant resilience to unfavourable conditions through a complex network of intertwined mechanisms (figure 2).

Microbes directly influence plant growth mainly by improving nutrient bioavailability. N is the most important nutrient for the plants as it plays a major role as part of amino acids, proteins and nucleic acids. Despite being the most abundant gas in the atmosphere, its availability for the plant uptake is often limited. Many PGPM contribute to N-fixing activity where they convert atmospheric nitrogen (N_2_) into ammonia (NH_3_), which is absorbed by the plant [85]. The presence of free-living N-fixer microbes like *Azospirillum* spp. in the rhizosphere uses root exudates as an energy source and enhance N bioavailability [86,87]. In addition, AMF are able to increase N-uptake through upregulation of nitrate reductase and therefore improve amino acid and protein biosynthesis (N use efficiency) [27]. Microbes that encourage root development are also indirectly linked to improved nutrient uptake as these plants can explore more soil surface [30,88].

The most well-known example of a symbiotic relationship is the rhizobium-legume interaction. In this association, *Rhizobium* forms nodules on the root of legumes where the ammonia is stored and is easily absorbed by the plant, which in return provides the bacteria with other metabolites, mainly sucrose [89]. *Rhizobium* is a popular inoculant; however, using other, free-living N-fixing species will provide bioavailable N to a wider range of crops [30]. Besides *Rhizobium*, the genera *Azospirillum, Pseudomonas, Bacillus, Azotobacter*, *Herbaspirillium*, *Arthrobacter, Azomonas, Clostridium, Corynebacterium, Derxia, Enterobacter, Klebsiella, Pseudomonas, Rhodospirillum, Rhodopseudomonas* and *Xanthobacterare* among others are also linked to N fixation in non-leguminous plants [85]. One of the primary goals of using microbial biostimulants is to enhance nutrient cycling. When aiming to reduce the use of chemical fertilizers, it is essential that a replacement (biostimulants) ensures sufficient levels of bioavailable N levels in the soil to maintain adequate crop yield.

Among microbes, microalgae and cyanobacteria are also integral components of the uppermost layer of the soil [90]. Although they are commonly associated with aquatic environments, there is now substantial evidence that algae are also part of the soil microbiome and the phytobiome. Many plant species exhibit symbiotic interactions with algae colonizing plant tissues similar to other symbiotic or mutualistic microorganisms. For instance, Lee & Ryu [91] reviewed the unique mechanisms by which Nostoc colonizes the roots of the angiosperm plant *Gunnera L*., fixing N and forming unique structures called glands. Although this process resembles the formation of tumours by *Agrobacterium* or nodules by *Rhizobium*, the molecular mechanisms of the *Nostoc-Gunnera* symbiotic interaction are unique to the presence of Nostoc [91].

Similar to N, phosphorus (P) and iron (Fe) are also abundant in soils but mostly in an insoluble form. PGPM play an important role in increasing the availability of P and Fe to plants. They achieve this by solubilizing insoluble P and Fe through the production of organic acids, which lower the soil pH, or by mineralizing organic P using extracellular enzymes [85]. One group of essential microbes known for enhancing P uptake in soils with low soluble P is AMF [92]. In addition, PGPM enhance Fe availability through Fe sequestration by the production of iron chelators known as siderophores (figure 2) [85]. This is again another important way for plants to retrieve essential nutrients from the soil that are not readily bioavailable.

Soil microbes produce a diverse array of bioactive compounds, including phytohormones such as auxin, cytokinin, gibberellic acid, precursors of ethylene (ETH) and abscisic acid (ABA). These signalling molecules actively regulate phytohormone homeostasis and influence processes related to plant growth and development. Auxins in the form of indole−3-acetic acid (IAA) produced by PGPM is able to regulate primary root growth and proliferation of lateral roots and root hair development [2]. Increased exposure to IAA therefore increases root growth and therefore the coverage of the root system [88,93–95]. Indirectly, this improves nutrient use efficiency as plants can explore more surface and reach deeper into the soil layers [23]. Next to IAA, cytokinins are also related to enhanced cell division and root development.

Lower levels of ETH, often referred to as the stress hormone, are associated with enhanced root growth [96]. ETH accumulation is regulated by the enzyme 1-aminocyclopropane−1-carboxylate (ACC) deaminase which can be produced by PGPM. ACC, an ETH precursor, is metabolized by ACC deaminase leading to a direct reduction in ETH production [34]. Exogenous ABA excreted by PGPM serves a dual role, acting both as a plant growth regulator and as a key factor in enhancing abiotic stress resilience [2,97]. Other compounds like amino acids and volatile organic compounds produced by PGPM have shown to contribute to the plant growth–promoting effect by modulating root growth and plant defence mechanisms [98–101]. Overall, as nutrient conversions and phytohormone production take place in PGPM, their metabolism is interconnected with many plants’ metabolic processes and subsequently affects plant growth. Additionally, these signalling molecules, especially auxin and cytokinin found in microalgae and cyanobacteria, regulate plant-algae and plant-microbe interactions, facilitate root colonization and activate the plant immunity, serving as essential components for the biostimulatory effect [91].

Microbes in the rhizosphere enhance plant nutrient availability and hormone production. Beyond these direct interactions, the presence of these microbes also indirectly influences plant physiology, contributing to overall plant health and development. For example, instead of influencing plant metabolism, PGPM can also shape the rhizosphere communities itself. PGPM can regulate the rhizosphere environment thereby inhibiting proliferation of phytopathogens through the production of antibiotic compounds, competition for nutrients and space, release of lytic enzymes and quorum sensing processes [102, 35]. Furthermore, the presence of PGPM in the rhizosphere prime plants for pathogen recognition, triggering induced systemic resistance, which enhances defence mechanisms against future infections in plants [103,104]. Overall, these plant-microbe interactions result in plants that are better resilient towards (a)biotic stressors.

Furthermore, many secondary metabolites produced by the microbes in the rhizosphere may not be recognizable by the plant, but could also interfere with molecular pathways, biosynthesis or gene expression that subsequently affects the plant growth and development [23]. For instance, PSs are polymeric carbohydrate macromolecules with complex structures that typically alleviate the negative effect of abiotic stresses by inducing the production of phenolic compounds, antioxidant molecules and reactive oxidant species (ROS) scavenging enzymes [105]. PSs produced by microbes, including microalgae, form a biofilm that enhances moisture retention, facilitates the recruitment of beneficial microbes and promotes their colonization on the rhizosphere of host plants [106].

The production of PSs is a significant characteristic of microalgae, accounting for up to 46% of their dry weight [76]. While PSs from microalgae have been extensively reviewed by many authors [107–110], this review aims to highlight the significant research conducted on their potential role as biostimulants and stress alleviation in plants. Several strains of microalgae produce diverse types of PSs that mediate plant growth and plant defence responses [111]. Consequently, application of microalgae extracts containing exopolysaccharides to the soil enhances levels of oxidizable carbon in the soil, boosts readily available soil organic carbon and increases the overall availability of nutrients in the topsoil [90]. Moreover, the production of PSs, and therefore the available carbon, can also serve as carbon source promoting the thriving of other beneficial PGPM [112].

In addition, rhizosphere activity and a high functionality are often linked with improved soil organic matter (SOM) [102,113–115]. Encouraging the health of the soil and its microbiome biodiversity is often associated with building SOM [116–118]. Owing to a lack of attention for SOM in cultivated soils, agricultural soil has a great potential to sequester carbon. There is a need to conserve and regenerate productive soils by maintaining and building SOM, thereby contributing to sustainable agriculture [119,120]. Soils with critically low SOM (<2%) are widespread on cultivated land. These soils can play a crucial role in carbon (C) sequestration, as they are far from their C saturation point and therefore accumulate and stabilize C quickly [121].

Microbial biostimulants, together with C-rich amendments like microalgae, are therefore a potential candidate in increasing SOM and therefore sequester CO_2_ on a large scale [90,122,123]. As a result, the value of regenerating agricultural soil to a soil with a functional microbiome and high in organic matter will not only benefit crop production but could simultaneously act as a sink for atmospheric CO_2_ from the crops photosynthetic carbon to the secreted root exudates in the soil and the conversion/use of these exudates by the rhizosphere microbiome [124–127].

Furthermore, applying complementary cultivation methods, such as soil management, crop rotation, the use of cover crops and carbon-based manure to maintain PGPM presence after inoculation, reduces the need for reapplication [128]. Next to that, prebiotic application is a noteworthy way of supporting PGPM activity in the soil to promote plant health as they have shown to improve soil ecology [129,130]. For instance, although there is no evidence of prebiotic activity, microalgae crude extracts contain bioactive compounds that can readily nourish the soil microbiome. The use of prebiotics would decrease the demand for required quantities and the strain production processes [61,131].

Regarding microalgae, their ability to produce many valuable components, along with their synergistic effect with other beneficial microbes, suggests that microalgae are good candidates for the engineering of synthetic communities (SynCom) that can effectively improve crop productivity [106,112]. The direct or indirect effect of algae on crops is still an emerging concept, and its study remains limited. Both the inoculation of live algae and the application of bioactive molecules derived from their crude extracts could be a good strategy to maintain soil structure, properties and functionality and increase stress resilience in plants [91,132].

## 4. Is abiotic stress alleviation in crops the biostimulants’ golden feature?

Given that plants are frequently exposed to diverse abiotic stresses, biostimulants provide a diverse range of specific molecules that aid in the biosynthesis of various stress-induced compounds like phytohormones, amino acids and sugars [42]. While specific mechanisms behind stress alleviation of biostimulants are still under investigation, PGPM share similarities with algae and cyanobacteria in mitigating environmental stresses (figure 2). They achieve this by producing bioactive metabolites to modulate pathways that regulate stress perception and signalling in plants [35]. In this section, we highlight the common effects of microbial and microalgae biostimulants on plants under abiotic stresses.

### (a) Phytohormones

In survival mode, plants experience a shift in phytohormone levels, characterized in general by an increase in ABA and ETH levels and a decrease in cytokinins and auxins. These communication/signalling pathways are species-specific and varied, allowing microbial biostimulants to target plant metabolism and enhance stress resilience. Owing to the large variety of mechanisms by which microbes communicate, they have been shown to influence all stress-related plant responses, including epigenetic modifications, transcription and translation (protein synthesis), phytohormone regulation, and ROS scavenging [27,133]. In this way, microbes can aid in the cry for help and allow plants to better withstand abiotic stress by maintaining growth, reducing damage and improving recovery [134].

ABA plays an essential role in abiotic stress and serves as an important link between the soil microbiome and enhanced drought stress resilience in plants. Inoculation with bacteria such as *Azospirillum* and *Pseudomonas* has been shown to stimulate ABA accumulation in *Arabidopsis thaliana*, tomato and maize both *in vitro* and in greenhouses. This, in turn, induces pathways that ultimately reduce water loss from the plants [97,135–138]. Microbial-induced ABA accumulation can regulate stomatal closure and chlorophyll biosynthesis, sustaining photosynthesis and osmotic adjustment, therefore improving water use efficiency and maintaining plant survival during drought stress [137]. Furthermore, inoculation with an auxin-producing *Bacillus* strain during drought conditions has been also shown to improve drought tolerance in tomato and wheat grown in greenhouse conditions [94,139,140]. PGPM inoculation was shown to enhance root phenotypes, including root biomass, elongation and root hair development [82]. Therefore, this modulation of the auxin and ABA signalling network by PGPM both improves water and nutrient uptake and water use efficiency from plants under drought stress.

Microalgae and cyanobacteria are also known for the production of growth-promoting and defence-related molecules such as auxin, cytokinin, gibberellins, ABA, jasmonic acid, salicylic acid and ETH [91,112]. Production of auxin by algae is crucial in the regulation of plant-algae [91] and microbe-algae interactions, colonization of host roots and activation of plant immunity [112]. Although there are no current studies on the role of algae-derived phytohormones in plant stress alleviation, they certainly play a critical role in regulating stress recovery in many plant species. For instance, to understand the importance of algae-derived cytokinins, Hussain *et al*. [141] generated a cytokinin knockout Nostoc mutant. This mutant showed a significant reduction on colonization of rice and wheat under axenic conditions, whereas the wild-type Nostoc enhanced phenotypic growth parameters of both plant species. Further research on algae-derived phytohormones is essential to understand their function in stress alleviation and their plant growth–promoting effects.

### (b) Osmolytes

In addition to hormone regulation, osmoregulation mechanisms are also affected by the presence of PGPM. The initial response of plants to drought stress involves modifying their osmotic potential by producing osmolytes and closing stomata to minimize water loss [134]. Numerous studies have confirmed that production of molecules such as proline (Pro), total soluble sugars and glycine-betaine is directly linked to reducing the adverse effects of osmotic stress [142–144]. These molecules enable plants to activate stress-responsive genes and initiate morphological adaptations [27,30].

While there is some debate about the correlation between Pro accumulation and stress response, this osmolyte plays a crucial role in the oxidative defence system by quenching ROS, thereby enhancing the robustness and activity of the oxidative defence. This effect, for example, has been shown in tobacco transgenic lines overproducing Pro owing to co-expression of rice genes for Pro biosynthesis (*OsP5CS1* and *OsP5CS2*) that showed reduced cellular oxidative damage and reduced ROS levels in plants under saline stress [145].

Moreover, Pro also stabilizes cellular structures such as membranes and buffers cellular redox potential under stress conditions. Pro accumulation in roots and leaves cells of plants under drought or salinity stress contributed to up to 50% of the osmotic adjustment [146]. The concentration of Pro in plants is correlated with stress tolerance; therefore, more sensitive plants have lower concentration of Pro under abiotic stress conditions [143]. An effective strategy to increase Pro levels in plants could be the exogenous supplementation of Pro or its precursors (l-glutamic acid), which can also enhance activity of enzymes with antioxidant capacity in plants under abiotic stresses [146].

Both inoculation of microbes and application of microalgae crude extracts induce plant physiology mechanisms for the production and accumulation of Pro in response to osmotic stress [144,147,148]. Also microalgae are known for naturally producing Pro as a protective response against abiotic stresses owing to their constant exposure to excess of salts, temperature fluctuations and desiccation throughout their life cycle [149]. Furthermore, the symbiotic interaction between plants and microbes increased expression of stress tolerance genes such as for the enzyme Pyrroline−5 carboxylate synthase (*P5CS*) related to Pro production [150]. Studies on tomato plants have shown reduced cellular damage and salinity stress alleviation through increased Pro production and upregulation of ROS scavenger genes [144,150].

Detecting biostimulant molecules that trigger the production of osmolytes like Pro in plants has become a standard practice to confirm their growth-promoting effect. However, the mechanisms by which biostimulants induce the production of these molecules in plants remain incomplete. Nevertheless, either small molecules like rutin (Rut) and gallic acid found in *Chlorella vulgaris* and *Nannochloropsis salina* [151] or complex structures like PSs found in *Dunaliella salina* [105] were able to increase tolerance to abiotic stress in moringa and tomato plants, respectively. Rut is a flavonoid that was shown to increase drought and osmotic tolerance in tobacco plants by the overexpression of chalcone synthase (*NtCHS*)*,* an enzyme involved in the biosynthesis of flavonoids [142,152]. The exogenous application of Ru to maize seedlings under osmotic stress increases the production of osmolytes molecules [142]. This confirms that molecules or compounds found in microalgae extracts are efficiently improving the production of osmolytes essential for the defence of plants against environmental stresses.

Similar to Rut, PSs also aid stress tolerance by enhancing the production of ROS-scavenging enzymes. Plant membrane receptors detect neutral sugars from PSs as microbial-associated molecular patterns inducing signalling pathways such as activation of Ca^2+^ influx and ROS-scavenging enzymes [76]. Because of their complex nature, the functional activity of PSs differs according to their monosaccharide composition, degree of sulfation and molecular weight (MW). The low MW PSs have the greatest effects owing to their high permeability [76,107,153].

## 5. Do plant biostimulants work as expected?

Over the years, claims about the growth-promoting effects of biostimulants have been largely depicted in studies based on bioassays evaluating the phenotypic traits of plants [154–159]. Certainly, phenotypic bioassays are of great importance in the screening process for optimizing the optimal dosage and marketing of biostimulants, as this is ultimately what customers/farmers are interested in. However, advancements in detection technologies have enabled scientists to gain deeper insights into the specific mechanisms and the composition of biostimulants. Although identifying every compound in biostimulants remains an almost impossible mission, the application of *omics* strategies in microbes and microalgae biotechnology, biorefinery and plant sciences such as transcriptomics, metabolomics and other molecular techniques will facilitate researchers to shed light on their modes of action. This knowledge will make the custom design of biostimulants possible: species dependent, soil quality or environmental stress dependent.

Omics approaches used in the field of microbial biostimulants have shed some light on the molecular mechanisms behind the phenotypic effects of PGPM on crops. With transcriptomic studies, genes involved in the plant-microbe crosstalk can be detected and discover the specific mechanisms that are involved in the interactions. For example, application of bioactive compounds filtrated from *Trichoderma* showed 984 differentially expressed genes in tomato in a greenhouse experiment. These genes showed a relationship to phytohormone homeostasis, antioxidant activity, phenylpropanoid biosynthesis and glutathione metabolism [160]. A similar study in pepper grown in field conditions combined 16S metagenomics with plant transcriptomics and metabolomics [161]. Therefore elucidating not only the genes regulated in the plants upon inoculation with *Streptomyces* but also related plant metabolite profiles and shifts in rhizosphere compositions, thereby providing a more comprehensive understanding of the plant-microbe interactions. In addition, omics studies have shown that there is a plant growth–promoting effect visible in various crops on a transcriptional, metabolic and proteomic level, paving the way to better predicting the biostimulatory effect of microbial inoculants [38,160–164].

Until now, limited studies in algae biostimulants have shown concluding results using omics approaches. For instance, elaborate studies using metabolomics profiling showed that PSs from diverse strains of microalgae change lipid, sterol and alkane profiles in tomato leaves [107,165,166]. However, the degree of change differs based on the microalgae strain. For instance, while *Dunaliella salina* MS002 enhanced peroxidase and β−1,3-glucanase enzymatic activities and polyphenols content, *Dunaliella salina* MS067 improved chitinase and phenylalanine ammonia lyase activities and protein content [166]. Although small molecules from microalgae extracts like polyamines and amino acids have been found to alleviate various stresses [147,167], their precise role and mode of action are yet to be discovered. Technological advances now favour the detection of smaller molecules and trace amounts of complex mixtures, potentially aiding in unravelling their role in plant defence mechanisms.

PGPM have been shown to interfere with and modulate almost all aspects of plant physiology. However, the plant is also able to actively shape the soil microbiome, and plant-microbe interactions are a two-way dialogue [168]. Host genotypic variation between and within species has been shown to lead to distinct microbial communities, along with phenotypic traits like root physiology and the plant metabolome [169,170]. Microbes could therefore be considered an essential part of healthy plant growth and a crucial extension of plant functioning and survival. Nevertheless, this interaction has been mostly overlooked in earlier decades of plant research, breeding and cultivation.

Recently, SynCom have been developed to mimic the structure of natural soil offering enhanced nutrient uptake, increased microbial stability, improved soil health and stress resilience [171]. Bacteria and fungi have been included in the design of the SynCom [150]. However, since microalgae is also part of the natural microbiota of the soil, research could focus on the addition of microalgae to SynComs. Additionally, it is also worth exploring the cultivation of microalgae communities and the co-cultivation of microalgae with beneficial bacteria that can also enhance their biomass productivity. Engineering microalgae can also be a useful resource for tailoring plant biostimulants or increase sustainability by producing high-quality products for feed, cosmetics or industry and making a multiproduct biorefinery. However, the regulations should also accompany the production of these valuable components.

### (a) Feasibility of the production of biostimulants using a sustainable approach: a microalgae case

As mentioned in this review, the sustainable production of microalgae biostimulants is linked to three main stages: microalgae growth, biostimulant production and application to crops. Interestingly, recent studies have shown the feasibility of the entire process, concluding that indeed this is a sustainable method to enhance agricultural productivity and purify waste streams from diverse sources. Evidence of this is the Life Cycle Assessment performed for the valorization of microalgae grown in piggery wastewater by Rojo *et al*. [172]. The authors found that the identification and further optimization of possible hotspots such as biomass harvesting and CO_2_ capture by using membranes would reduce the environmental impact up to 30 and 16%, respectively. However, the same studies suggest that the effect of the long-distance transportation (longer than 321 km) of the wastewater to the microalgae treatment plant would imply an increased negative environmental impact. A solution to this could be the implementation of a microalgae treatment plant next to the wastewater source. In this way, the water can be purified and the impact on transportation will be drastically reduced.

There is no scientific evidence for the impact of the implementation of this method. Nevertheless, the European Union Commission is making efforts through the REALM project, which is currently investigating the feasibility of using microalgae for nutrient recovery from drain water from soilless greenhouses. In addition, the project also aims to revalorize the obtained biomass by further developing biostimulants to reduce the impact of chemical fertilizers in agriculture [173].

### (b) Potential of biostimulants to replace chemical fertilizers in modern agriculture

Besides carbon sequestration, one of the most common claims about biostimulants is to reduce chemical fertilizer by substituting portions of it in the current nutrient management practices. A one-size-fits-all approach and solely focusing on a single solution are probably not applicable in the case of biostimulants use in agriculture. However, actual tangible data to prove this concept are rare. Nevertheless, microbial and microalgae biostimulants, especially cyanobacteria, have been shown to improve nutrient use efficiency of chemical fertilizers [84,112,174,175] probably owing to their ability to increase the bioavailability of nutrients in the soil [63,176]. This feature of biostimulants can directly lead to lower concentrations of applied fertilizers and reduced run-off to the environment. Thus, biostimulants can partially replace some of the chemical fertilizers used.

In a recent study, combining a commercial microbial consortium with organic fertilizer allowed for a 50% reduction in chemical fertilizer while preserving plant biomass production of *Tagetes patula* [177]. Field-grown wheat showed increased plant biomass and yield when 20% of the chemical fertilizer was replaced with organic fertilizer supplemented by a microbial consortium, compared to using chemical fertilizer alone [63]. Cyanobacteria such as Nostoc and Anabaena were shown to contribute up to 25–50% of inorganic fertilizer reduction in spinach field trials [112,178]. However, complete replacement of inorganic fertilizers while maintaining the same crop yield may be unrealistic or too ambitious [43].

In addition, microbial biostimulants require time to establish and integrate into the existing microbiome in the fields, in order to exert a visible effect on the crops. This process may even take years instead of weeks, while chemical fertilizers have an immediate effect and can fix the soil nutrient status rapidly. However, in the long term, inorganic fertilizers are polluting the environment and pushing out the biodiversity of species including microbial biodiversity in the soil [179,180].

More research in the role of microbial biostimulants in field conditions is needed, as well as the development of optimal formulations, application methods and a deeper understanding of their underlying molecular mechanisms. Although more studies on commercial biostimulants are needed [28], there is huge variation in the microbes used, the quantity applied and the application methods employed. This variability makes comparisons between trials difficult [61]. Furthermore, the highest effectiveness of biostimulants tends to be there when addressing specific limitations in the soil or challenging environmental conditions.

The functionality of an inoculant is also often strain and host-plant specific, showing the importance of smart strain selection and design of the biostimulants. Even closely related microbial species may not be suitable for inclusion in biostimulants, as their effects can vary widely depending on their functions. Therefore, directly comparing chemical fertilizers with microbial biostimulants remains challenging, and the predictability of biostimulants has yet to be fully verified. Although the possibilities with biostimulants for agriculture seem endless, serving as a standalone solution for cultivation challenges is unlikely. Instead, together with appropriate cultivation methods, biostimulants have the potential to enhance crop health and productivity, offering a promising tool for sustainable agriculture.

## 6. Conclusions

In this review, we compile all the current and relevant information on the effectiveness of biostimulants and challenges on their development. Both microbial and microalgae crude extracts directly or indirectly stimulate plant growth using intricate molecular mechanisms that are yet to be understood. Elucidating the response of biostimulants through fundamental and applied research using state-of-the-art tools like omics approaches would not only enhance our understanding but also enable the development of more tailored-made biostimulants.

Using recycled sources such as agricultural waste or wastewater as substrates for the production of biostimulants will significantly enhance their sustainability. Thus, biostimulants have the potential to become an effective and environmentally friendly solution in the future. Moreover, although biostimulants enhance nutrient uptake of crops, they are not a replacement for chemical fertilizers, but rather a complement to optimize nutrient management practices, prevent soil degradation and increase resilience to abiotic stresses. Furthermore, using a combination of microbial and non-microbial biostimulants will contribute to the long-term soil health, as biostimulants are not an immediate remedy. However, they are still a novel and promising tool that needs to be refined in terms of standardization of their development and regulations.

## Data Availability

This article has no additional data.
